# Characterization of Fragile X Mental Retardation Protein Recruitment and Dynamics in *Drosophila* Stress Granules

**DOI:** 10.1371/journal.pone.0055342

**Published:** 2013-02-07

**Authors:** Cristina Gareau, Elise Houssin, David Martel, Laetitia Coudert, Samia Mellaoui, Marc-Etienne Huot, Patrick Laprise, Rachid Mazroui

**Affiliations:** Department of Molecular Biology, Medical Biochemistry, and Pathology, Faculty of Medicine, Laval University, Centre de recherche le CHU de Quebec, Quebec, Canada; Hertie Institute for Clinical Brain Research and German Center for Neurodegenerative Diseases, Germany

## Abstract

The RNA-binding protein Fragile X Mental Retardation (FMRP) is an evolutionarily conserved protein that is particularly abundant in the brain due to its high expression in neurons. FMRP deficiency causes fragile X mental retardation syndrome. In neurons, FMRP controls the translation of target mRNAs in part by promoting dynamic transport in and out neuronal RNA granules. We and others have previously shown that upon stress, mammalian FMRP dissociates from translating polysomes to localize into neuronal-like granules termed stress granules (SG). This localization of FMRP in SG is conserved in *Drosophila*. Whether FMRP plays a key role in SG formation, how FMRP is recruited into SG, and whether its association with SG is dynamic are currently unknown. In contrast with mammalian FMRP, which has two paralog proteins, *Drosophila* FMR1 (dFMRP) is encoded by a single gene that has no paralog. Using this genetically simple model, we assessed the role of dFMRP in SG formation and defined the determinants required for its recruitment in SG as well as its dynamics in SG. We show that dFMRP is dispensable for SG formation *in vitro* and *ex vivo*. FRAP experiments showed that dFMRP shuttles in and out SG. The shuttling activity of dFMRP is mediated by a protein-protein interaction domain located at the N-terminus of the protein. This domain is, however, dispensable for the localization of dFMRP in SG. This localization of dFMRP in SG requires the KH and RGG motifs which are known to mediate RNA binding, as well as the C-terminal glutamine/asparagine rich domain. Our studies thus suggest that the mechanisms controlling the recruitment of FMRP into SG and those that promote its shuttling between granules and the cytosol are uncoupled. To our knowledge, this is the first demonstration of the regulated shuttling activity of a SG component between RNA granules and the cytosol.

## Introduction

The RNA-binding protein Fragile X Mental Retardation (FMRP) is an evolutionarily conserved protein that is particularly abundant in the brain due to its high expression in neurons [1], [2], [3]. The absence of FMRP causes the development of Fragile X syndrome, the most frequent form of hereditary mental retardation [4], [5]. FMRP is considered to be a nucleocytoplasmic shuttling protein [6], [7], [8], [9]. In the cytoplasm, the major fraction of FMRP is associated with mRNP complexes bound to polyribosomes [10], [11], [12], in support of a translational role for FMRP [5], [13], [14], [15]. In neurons, FMRP may also act as a translational repressor by trapping mRNAs into neuronal RNA granules which are then transported out of the soma in a repressed state until they reach their destination in the neurites [13]. It was previously suggested that mammalian FMRP might also promote translation repression of its mRNA targets under stress conditions by trapping them into stress granules (SG) [16]. SG are cytoplasmic bodies whose formation during stress correlates with the inhibition of translation initiation and might constitute the actual sites where stalled translation initiation complexes accumulate [17], [18]. The formation of SG, which occurs under stress conditions, requires the phosphorylation of eIF2α, a key pathway known to induce translation initiation arrest upon stress [19]. SG formation may also occur by inactivation of other translation initiation pathways independently of eIF2α phosphorylation [20], [21]. A recent study identified FMRP as a potential SG-promoting factor in mammalian cells, although the underlying mechanism is still undefined [22]. In addition to FMRP, mammalian genomes encode two other members of this family, namely FXR1 and FXR2 [23], with which they do co-localize together with FMRP in stress-induced SG [16]. On the other hand, *Drosophila* encodes only one member of the FMRP family, i.e. dFMRP [24]. dFMRP shares the basic molecular functional determinants with its mammalian homologues, implying a conservation of FMRP functions between flies and mammals [24]. These conserved domains include the N-terminal protein-protein domain which is known to promote FMRP dimerization and interactions with its partners, as well as KH and the RGG box, which act as RNA-binding motifs [7]. The C-terminal region of dFMRP is a highly glutamine and asparagine (Q/N)-enriched domain, which facilitates protein interactions [25]. Likewise mammalian FMRP, dFMRP do accumulate in SG upon stress [26].

In the present study, we investigated the role of dFMRP in SG formation and defined the determinants required for the accumulation of dFMRP in SG as well as those that are required for its dynamics in and out SG. We found that decreasing dFMRP levels in *Drosophila* Schneider cell does not prevent SG formation upon either arsenite or heat shock, and we recapitulated these results using ovaries isolated from *dfmr1*-null flies. Using live cell imaging, we show that both KH and RGG domains, as well as the C-terminus polyQ/N are required for dFMRP localization SG. The protein-protein interaction domain located at the N-terminal part of dFMRP is dispensable for such localization. This protein-protein interaction domain of dFMRP is however required for the dynamic trafficking of dFMRP between SG and the cytosol. The kinetics of the shuttling activity in both SG and dFMRP granules are thus conserved between flies and mammals.

## Results

### Stress Induces the Release of dFMRP from Dissociating Polysomes and its Accumulation in SG in *Drosophila* Cells

**Figure 1:**
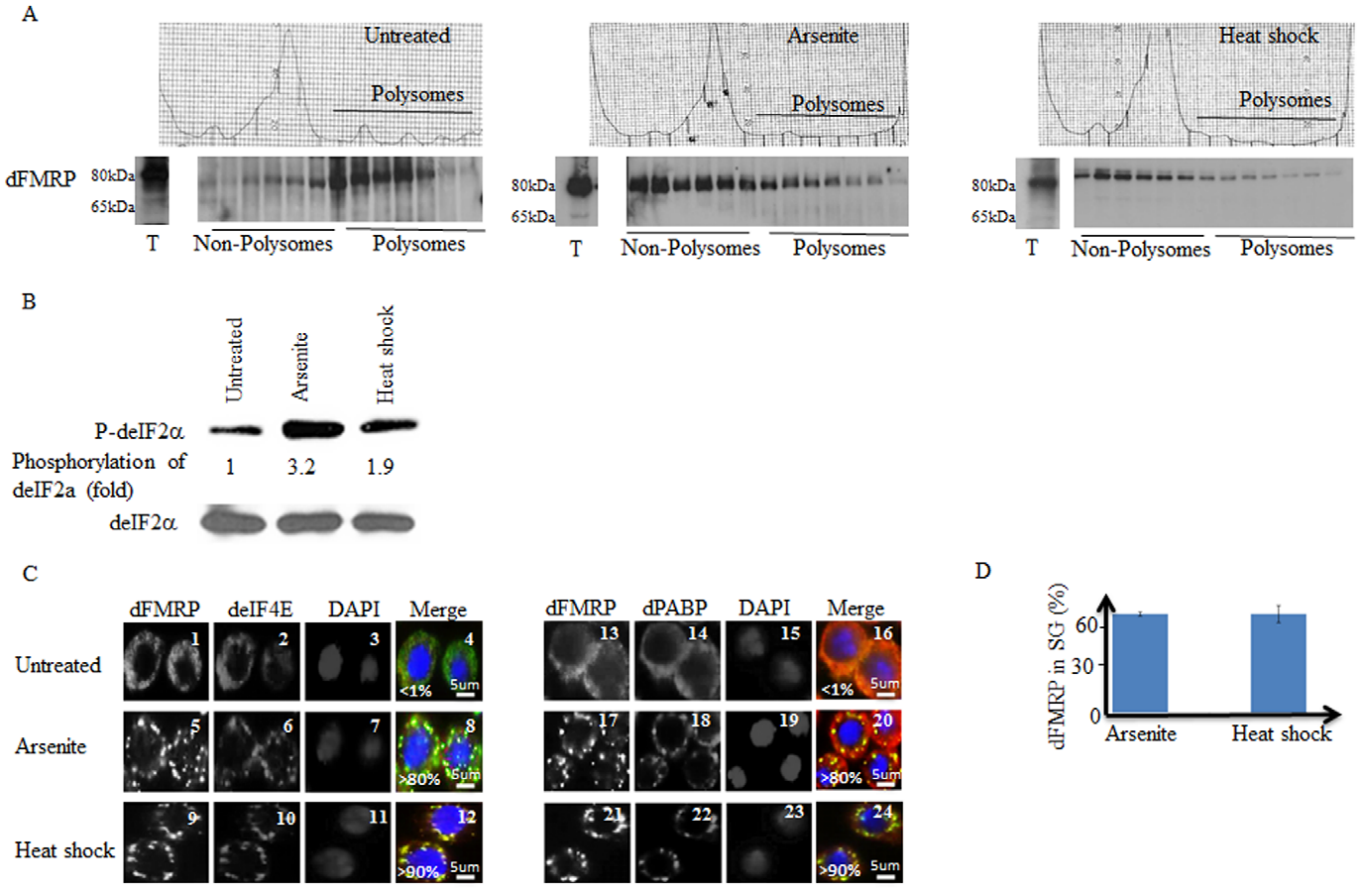
Stress induces partial translocation of dFMRP from dissociating polysomes and its accumulation in SG. (A–C) Sucrose gradient analysis of polysomes and analysis of dFMRP distribution on polysomes. (A) Schneider cells were untreated (left panels) or treated either with arsenite (0.5 mM; center panel) or heat shock at 37°C (right panel) for 1.5 h. Cytoplasmic extracts were sedimented through sucrose density gradients and dFMRP distribution was analyzed by western blot using specific antibodies. (B) eIF2α phosphorylation. Schneider cells were treated with arsenite (0.5 mM) or incubated under heat shock conditions (37°C) for 1.5 h. Total cell lysates were then prepared and analyzed by western blot for the phosphorylation of eIF2α using specific antibodies. Total eIF2α was analyzed using the pan-eIF2α antibodies. The amount of phosphorylated eIF2α was determined by quantitation of the film signals by densitometry using the Adobe Photoshop and expressed as a percentage of total eIF2α. The results are representative of 5 different experiments. (C–D) Schneider cells were treated with 0.5 mM arsenite or heat shock (37°C) for 1.5 h, fixed, permeabilized, and processed for immunofluorescence using antibodies against different SG markers: dPABP and deIF4E; (red signal in merged pictures) and dFMRP (green signal in merged pictures). DAPI (blue signal in merged pictures) is used as a nuclear stain. Pictures were taken using a 63X objective at 1.5 zoom (C). The percentage of cells harboring SG (>3 granules/cell) from 5 different fields and 5 different experiments containing a total of 2,000 cells is indicated at the bottom of merged images. Scale bars are indicated. (D) Densitometry of dFMRP immunofluorescence signal in SG with Adobe Photoshop. The number of pixels and mean intensities were recorded for the selected regions (SG, cytoplasm and background) using Photoshop. The mean intensity was multiplied by the number of pixels for the region selected to obtain the absolute intensity. The absolute intensity of the background region was subtracted from each region of interest. To compare the intensity between two given regions of interest, relative intensities were next calculated. Relative intensities correspond to the absolute intensities normalized to the absolute intensity of the region of reference.

It was previously shown that treatment of Schneider cells with either arsenite or heat shock induces dFMRP accumulation in SG, which correlates with polysome dissociation [26]. Since the major fraction of FMRP is known to associate with polysomes, we assessed whether accumulation of the protein in SG in *Drosophila* cells is due to dissociation of polysomes during stress. First, we assessed polysome profiles of Schneider cells treated with either arsenite or heat shock. As shown in Fig. 1A (center and right top panels), both types of stress induce a large decrease of polysome peaks concomitant with an increase of the 80S peak, indicating an inhibition of translation initiation. This translational block was further demonstrated by assessing eIF2α phosphorylation, which was significantly induced by either arsenite or heat shock (Fig. 1B). We then determined if polysomes dissociation that is triggered by arsenite and heat shock induces loss of dFMRP from polysomes fractions. As expected, control experiments showed that dFMRP is distributed mainly at polysomes fractions in untreated cells (left bottom panel). Treatment with either arsenite (center bottom panel) or heat shock (right bottom panel) reduced the amount of dFMRP found at polysome fractions (Fig. 1A). This result indicates that stress-mediated dissociation of polysomes leads to the release of dFMRP from dissociating polysomes. As previously documented [26], treatment of Schneider cells with either arsenite (Fig. 1C; panels 5–8 and 17–20) or heat shock (Fig. 1C; panels 9–12 and 21–24) induced SG, in which dFMRP co-localizes with the canonical SG markers deIF4E (Fig. 1C; panels 8 and 12), dPABP (Fig. 1C; panels 20 and 24 ), GFP-eIF4A and with poly(A)+mRNA (Fig. S1; panel 8 and data not shown). Our quantification studies showed that over 90% of dFMRP-containing SG are also positive for the other SG markers (data not shown). These SG are reversible as they disassemble during the recovery phase from stress (Fig. S2; compare panel 4 with panel 7 and panel 10 with 13). Our quantification of dFMRP signal revealed that a significant fraction (∼60%) of total dFMRP localizes in SG upon either arsenite or heat shock (Fig. 1D), which as described above correlates with dFMRP releases from dissociating polysomes (Fig. 1A). This indicates that stress conditions induce the recruitment of dFMRP from dissociating polysomes to accumulate in SG. These results do not exclude however the possibility that dFMRP present in non-polysomal fraction can also be recruited in SG. The apparent accumulation of dFMRP in non-polysomal fractions under stress conditions (Fig. 1A; center and right bottom panels) is likely due to the high shuttling activity of dFMRP between SG and cytosol (see below). We conclude that the recruitment of FMRP from dissociating polysomes into SG is conserved in flies.

### dFMRP Depletion does not Alter SG Formation

**Figure 2:**
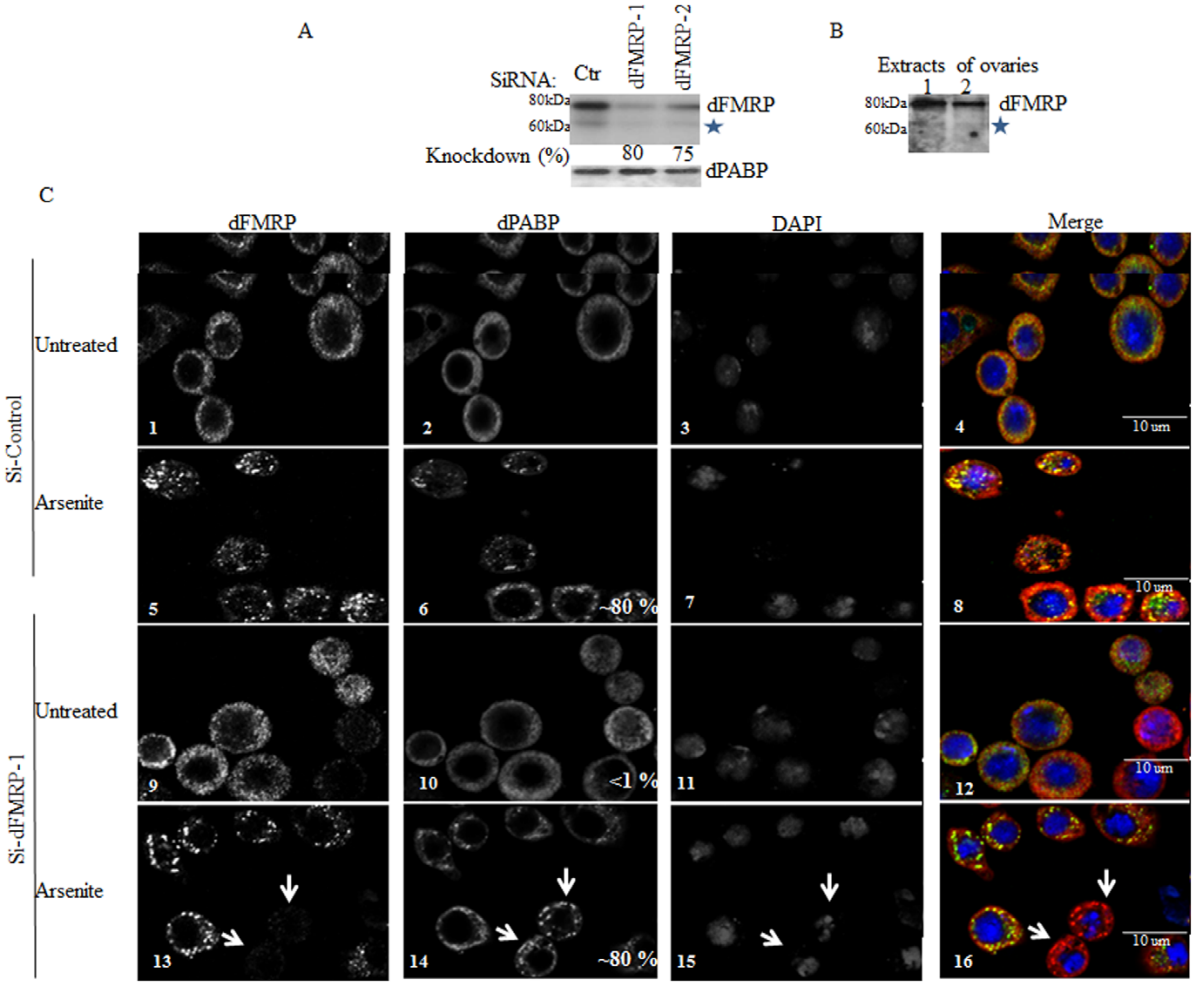
Reducing dFMRP levels has no effect on SG formation. (A) Schneider cells were treated with non-specific or dFMRP-selective siRNAs 1 and 2 for 96 h. Cells were lysed and protein extracts were prepared and analyzed by western blot to detect dFMRP and dPABP proteins (loading standards) using the appropriate antibodies. The percentage of dFMRP knockdown was determined by quantification of the film signal by densitometry using Photoshop and expressed as a percentage of total dPABP. Asterix denotes a lower migrating band reacting with anti-dFMRP antibodies. (B) Ovaries extracts were prepared and analysed by western blot for dFMRP expression using anti-dFMRP antibodies. Asterix denotes a lower migrating band reacting with anti-dFMRP antibodies. (C) Schneider cells were treated with non-specific or dFMRP-selective siRNAs 1 and 2 for 96 h. Cells were treated with arsenite (0.5 mM) for 1.5 h and then processed for immunofluorescence as described in Fig. 1 using anti-dFMRP (green signal in merged pictures) and anti-dPABP (red signal in merged pictures) antibodies. DAPI is used as a nuclear stain. Pictures were taken using a 63X objective. Scale bars are indicated. The percentage of cells harboring SG (indicated at the bottom of dPABP images) was calculated as in Fig. 1. Arrows depict dFMRP-depleted cells. Note that the percentages of SG in dFMRP-depleted cells and mock-depleted cells are similar.

Similarly to the situation described above in *Drosophila* cells, we and others have shown that treatment of mammalian cells with arsenite induces localization of FMRP in SG, which correlates with its dissociation from polysomes [16], [27]. In a more recent study, it was shown that mouse embryonic fibroblasts (MEF) cells, which lack endogenous FMRP, fail to form SG efficiently [22]. This suggests that recruitment of FMRP from dissociating polysomes and its subsequent association with SG could promote their stabilization, thus enhancing their formation. We thus investigated the role of dFMRP in SG formation. Schneider cells were treated with two specific dFMRP1-siRNAs to deplete dFMRP and SG formation was assessed in dFMRP-depleted cells following treatment with either arsenite (Fig. 2) or heat shock (data not shown). Western blot analysis shows that dFMRP was efficiently depleted (∼75%) by treatment with either dFMRP1-siRNAs (Fig 2A). Our western blots analysis detected a lower minor band migrating at ∼65 KDa, which was recognized by anti-dFMRP antibodies. A similar lower migrating band (indicated by asterix) was also detected in *Drosophila* ovaries extracts using anti-dFMRP antibodies (Fig. 2B). This detected band likely corresponds to the previously described minor isoform of dFMRP lacking the C–terminus (see below). Immunofluorescence experiments showed that depletion of dFMRP (which was confirmed with specific antibodies) did not affect SG formation as detected by antibodies against the two SG markers dPABP (Fig 2C, panels 13–16; dFMRP-depleted cells are indicated by arrows) and deIF4E (data not shown). Localization of the SG marker GFP-deIF4A in SG was also evident in dFMRP-depleted cells upon treatment with either arsenite (Fig. S3, panels 13–16; dFMRP-depleted cells are indicated by arrows) or heat shock (data not shown), further indicating that SG formation can still occur under conditions of low dFMRP protein levels.

**Figure 3:**
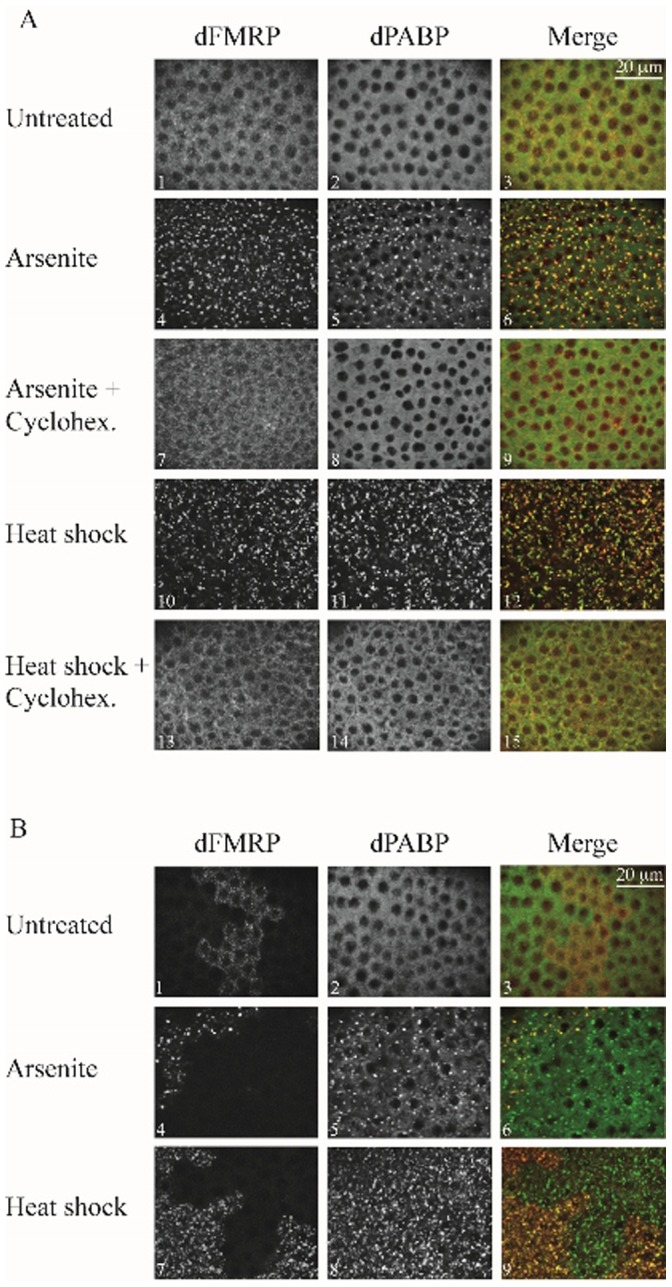
SG formation in Drosophila ovaries is not affected by dFMRP deficiency. (A) Ovaries isolated from WT flies were treated with cycloheximide (100 µg/ml) for 0.5 h then were either heat-shocked at 37°C for 3 h or incubated with 0. 5 mM arsenite for 1.5 h, in presence of cycloheximide. Ovaries were then fixed, permeabilized and processed for immunofluorescence as described in “Materials and methods”. SG were visualized using both anti-dFMRP and anti-dPABP antibodies. Note that both heat shock and arsenite induces SG formation according to a process that is prevented by the addition of cycloheximide. (B) The formation of SG was assessed in *Drosophila* ovaries harboring clonal dFMRP-knockout cells and cells expressing dFMRP. Ovaries were treated with either heat shock for 3 h at 37°C or incubated with 0. 5 mM arsenite for 1.5 h, permeabilized and processed for immunofluorescence. SG formation was assessed using anti-dPABP antibodies. Anti-dFMRP serves to distinguish between dFMRP-positive and -negative clones in the analyzed ovaries. We observed at least 20 clones for each condition, and the phenotype is penetrant at 100%. Scale bars in A and B are indicated.

We then attempted to reproduce the latter results by investigating SG formation in *dfmr1*-null flies. To our best knowledge, formation of SG has never been previously documented in *Drosophila* tissues. Thus, we first sought to characterize SG formation in *Drosophila* ovaries *ex vivo*. For these experiments, we used heat shock as an SG inducer because heat shock conditions have been well established in fruit flies, and then validated our results using arsenite. As shown in Fig. 3A, treatment of ovaries isolated from wild-type (WT) adult flies with either heat shock (panels 10–12) or arsenite (panels 4–6) induces granules that are positive for both dFMRP and dPABP. These heat shock-induced granules are not detected in untreated samples (Fig. 3A; panels 1–3). SG formation is known to be prevented in stressed cells upon treatment with translation elongation inhibitors such as cycloheximide, which results in mRNA “freezing” on translating polysomes [28], [29] (see also Fig. S4A; compare panels 4 and 6 with 7 and 9). In contrast, puromycin, a component that induces polysomes disassembly by promoting premature termination, does not inhibit formation of SG in stressed cells [28], [30] (see also Fig. S4B; compare panels 4 and 6 with 7 and 9). As expected, control experiments show that puromycin preserved SG formation in both heat-shocked and arsenite-treated ovaries (Fig. S5A). In contrary, cycloheximide treatment of isolated ovaries prevented granule formation in either heat-shocked or arsenite-treated ovaries (Fig. 3A; compare panels 4–6 with 7–9, and panels 10–12 with 13–15), thus validating the identification of these granules as SG. Next, we used ovaries harboring homozygous dFMRP mutant clones (Fig. S5B) to assess SG formation upon either heat shock or arsenite treatment (Fig. 3B). SG formation was similarly induced in both dFMRP1-positive and -negative clones, as assessed by the localization of dPABP (Fig. 3B, panels 4–9), indicating that dFMRP deficiency in *Drosophila* ovaries does not alter SG induction by either heat shock or arsenite treatment. Our results thus show that dFMRP is not absolutely required for SG formation in flies tissue tested here, corroborating our results obtained *in vitro*.

### Characterization of dFMRP Recruitment in SG

**Figure 4:**
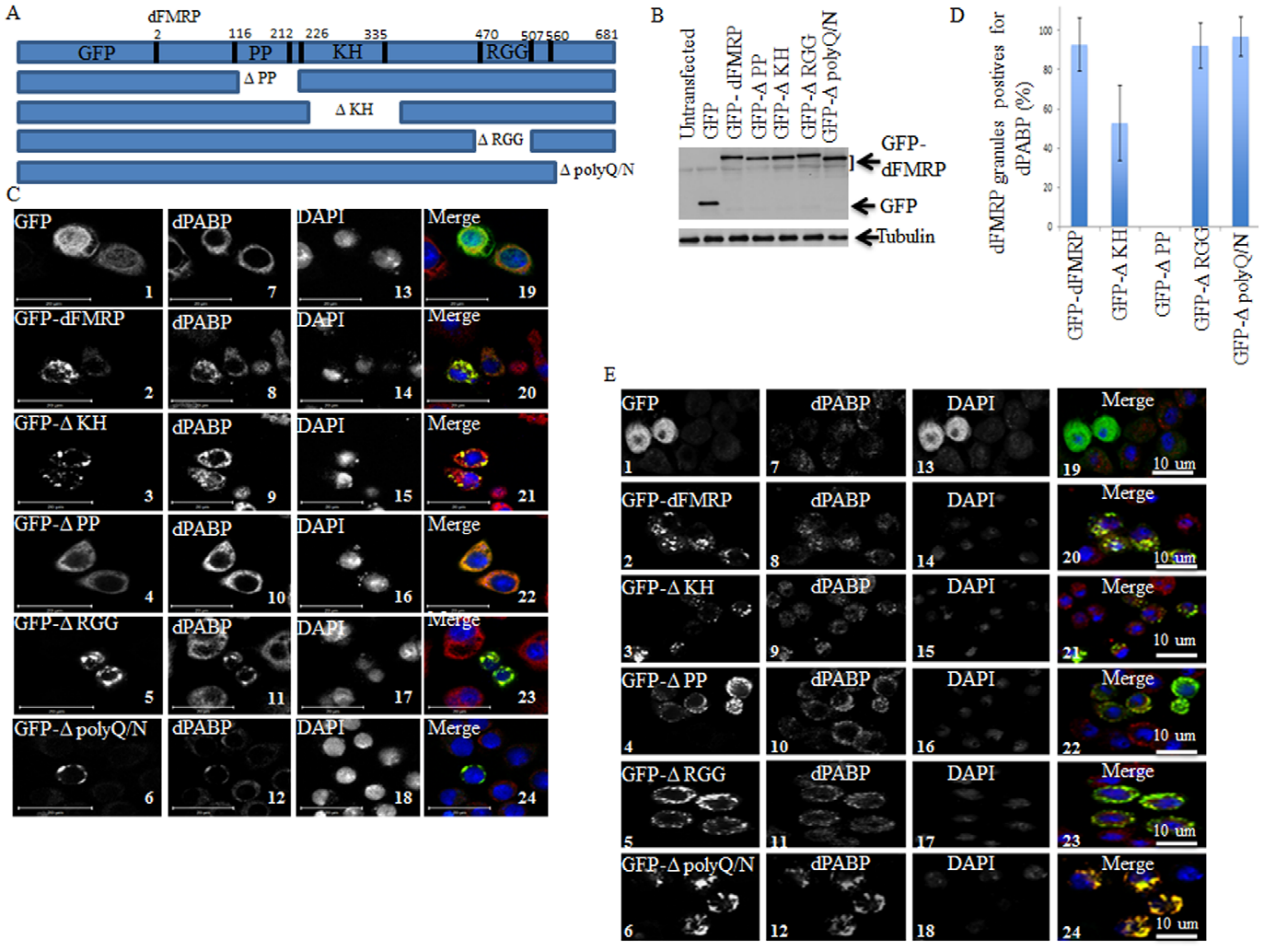
Localization of GFP-dFMRP fusion proteins in SG as analyzed in fixed cells. (A) Schematic representation of GFP-dFMRP (top) and its deletion versions. (B) Schneider cells were transfected with either GFP or GFP-dFMRP constructs for 48 h. Cells-expressing GFP-dFMRP were then collected and protein extracts were next analyzed by immunoblotting for GFP-dFMRP expression using anti-GFP antibodies. Tubulin was used as a loading control. (C–D) Schneider cells were transfected with either GFP or GFP-dFMRP constructs for 48 h. Cells were then fixed and then processed for immunofluorescence to detect GFP or GFP-dFMRP (*green*). The intracellular localization of endogenous dPABP (C; *red*) is revealed using antibodies specific to dPABP. The indicated percentage of dFMRP-granules harboring dPABP (D) is calculated from 3 different experiments containing a total of 500 transfected cells. Scale bars are indicated. (E) Schneider cells were transfected with either GFP or GFP-dFMRP constructs for 48 h. Cells were then treated with arsenite (0.5 mM; 1.5 h), fixed and processed for immunofluorescence to detect dPABP using specific antibodies (red signal). GFP and GFP-dFMRP are detected as green fluorescence. Scale bars are indicated.

**Figure 5:**
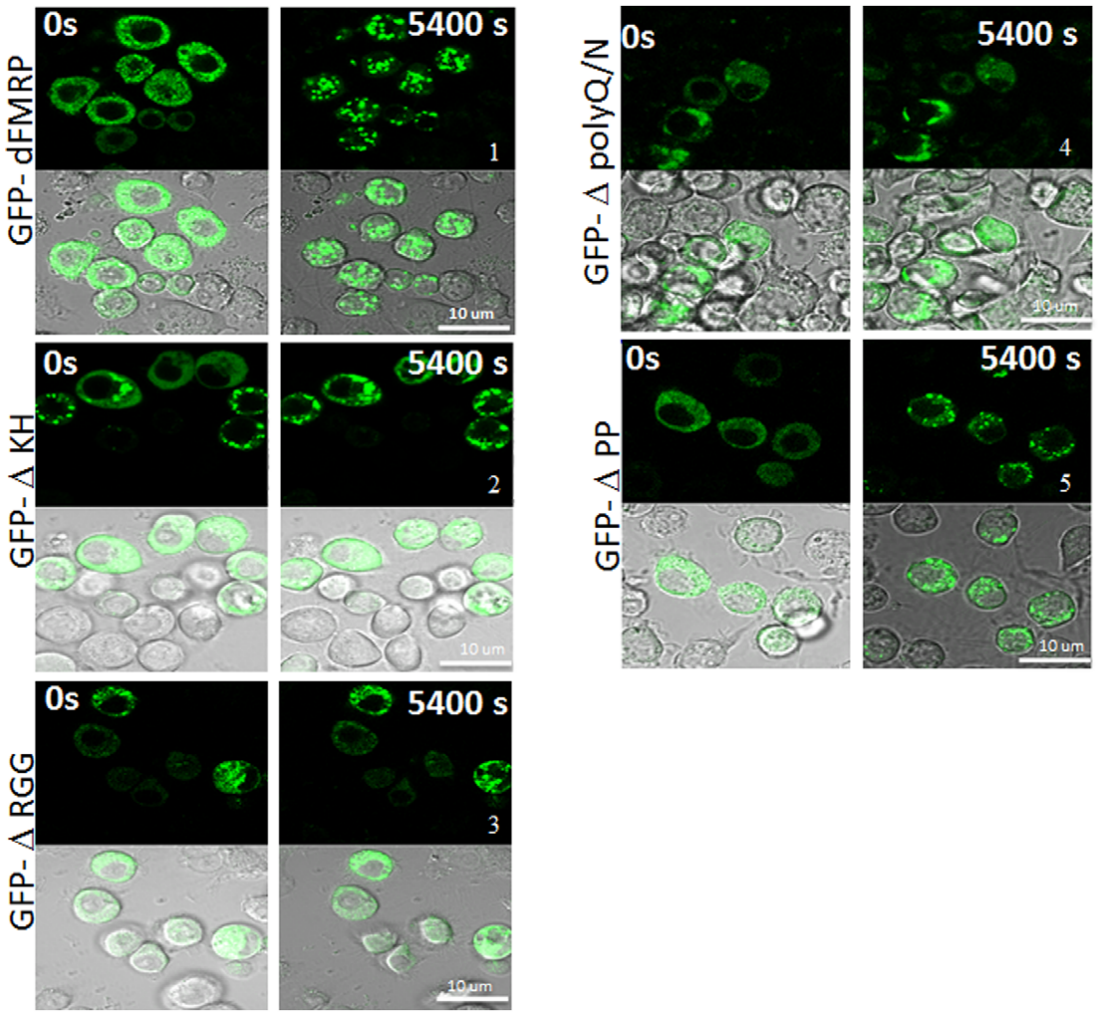
Localization of GFP-dFMRP fusion proteins in SG as visualized in live cells. Schneider cells were transfected with GFP-dFMRP fusion proteins. After 48 h, SG were induced with arsenite (0.5 mM) and cells were observed in live by confocal microscopy over 1.5 h. Image acquisitions were taken every 3 min. The same cells are shown for GFP-dFMRP protein fluorescence (top panels) and DIC (bottom panels), at zero and 1.5 h. Scale bars are indicated.

Our results described above (Fig. 1C–D) show that dFMRP is quantitatively recruited in SG. How FMRP is recruited into SG is still unknown. To address this question, we investigated the contribution of each domain of dFMRP in its recruitment into SG. For these experiments, we constructed several GFP-dFMRP versions in which each known conserved domain has been selectively deleted, leaving the rest of the protein intact (Fig. 4A). ΔPP refers to dFMRP lacking the Protein-Protein interaction domain (116–212) located at the N-terminal region of the protein. ΔKH lacks the conserved KH domain at positions 226–335, and ΔRGG is a construct lacking the RGG box (470–507). ΔpolyQ/N is a mutant lacking the C-terminal polyglutamine-asparagine rich region, thus mimicking the splice variant of dFMRP which naturally lacks the C-terminus [25]. Schneider cells were transfected with GFP-dFMRP, treated with arsenite, and the localization of GFP fusion proteins in SG was then visualized. All mutants are well expressed as assessed by both western blot analysis of GFP-dFMRP using anti-GFP antibodies (Fig. 4B) and by fluorescence to detect GFP as green fluorescence (Fig. 4C). Control experiments show an expected cytoplasmic localization of GFP-dFMRP under normal growth conditions (Fig. 4C; panel 2). However, and as previously documented in mammalian cells [16], [31], expression of GFP-dFMRP induced formation of SG-like cytoplasmic granules (named dFMRP granules) in >50% of transfected cells, in absence of any additional stress (Fig. 4C; panel 2). These dFMRP granules are positive for the two SG markers dPABP (Figs. 4C; panels 8 and 20) and deIF4E (Figs. S6; panels 1–2). Expression of either ΔKH (Fig. 4C; panel 3), or ΔRGG (Fig. 4C; panel 5), or ΔpolyQ/N (Fig. 4C; panel 6) mutants induces the formation of dFMRP granules in >50% of transfected cells. These dFMRP granules are positives for both SG markers dPABP (Figs. 4C; panels 21 and 23–24), and deIF4E (Fig. S6; panels 3–4 and 7–10). The percentage of dFMRP-granules harboring dPABP (Fig. 4D) and deIF4E (data not shown) is significantly high (50–90%), suggesting a possible role of these granules in sequestering SG markers. Finally, expression of ΔPP does not induce dFMRP granules (panels 4 and 22 of Fig. 4C, and panels 5–6 of Fig. S6). Arsenite induced SG in most untransfected cells analyzed, as judged by the granular co-localization of dFMRP with SG markers dPABP and deIF4E (Figs. 4E and S7; see also Fig. 1D). Under those arsenite conditions, GFP-dFMRP and its mutant’s ΔKH, ΔRGG and ΔpolyQ/N do localize in granules that are positive for both dPABP (Fig. 4E; panels 21 and 23–24) and deIF4E (Fig. S7) in most transfected cells. However, these localization studies in fixed cells could not determine whether the detected granules represent arsenite-induced SG or merely preformed dFMRP granules that were induced by GFP-dFMRP expression before arsenite addition. It is important to distinguish between these two possibilities in order to determine if expressed GFP-dFMRP proteins are incorporated in newly formed SG induced by stress. More clear results were obtained using the ΔPP mutant, which as described earlier, does not induce dFMRP granules. Rather, ΔPP is uniformly distributed in the cytosol of unstressed cells (Fig. 4C; panel 4). We found that this mutant is efficiently recruited in arsenite-induced SG as assessed by its co-localization with both dPABP (Fig. 4E; panel 22) and deIF4E (Fig. S7). This result established that the PP domain of dFMRP is dispensable for localization of the protein in arsenite-induced SG. The above-described results (Figs. 4E and S7) also established the ΔPP mutant as an appropriate SG marker to assess SG induction by stress and to visualize SG without inducing dFMRP granule formation. Overall, the results obtained using fixed cells suggest that dFMRP-protein interactions mediated by the PP domain are dispensable for its localization in SG. Because expression of either ΔKH, or ΔRGG, or ΔpolyQ/N mutants induces formation of dFMRP granules, we could not conclusively investigate their recruitment in SG using fixed cells. Therefore, to ascertain whether or not GFP-dFMRP is recruited in SG, we monitored changes in their distribution in live Schneider cells upon arsenite addition (Fig. 5 and Videos S1, S2, S3, and S4). First, we assessed the recruitment of GFP-WT-dFMRP in SG. Since the expression of GFP-dFMRP can induce dFMRP granules, we attempted to monitor the localization of proteins in selected cells containing preformed dFMRP granules, as well as in cells lacking such preformed dFMRP granules before arsenite treatment. This is important in order to assess whether preformed dFMRP-induced granules can affect the recruitment of GFP-dFMRP in SG. We found that arsenite treatment of cells that are devoid of preformed dFMRP granules rapidly (≤15 min) induces the recruitment of GFP-dFMRP in SG (Fig. 5; panel 1 and Video S1). We also noticed that the distribution of GFP-dFMRP in cells containing preformed dFMRP granules did not significantly change upon arsenite treatment (Video S1). These results suggest that while cytosolic GFP-dFMRP may be transferred to SG induced by arsenite, GFP-dFMRP present in dFMRP granules did not shift its localization under similar stress conditions. We speculate that GFP-dFMRP present in dFMRP granules cannot be recruited to SG, although we do not have direct evidence that dFMRP granules and SG are distinct RNA granules. However, one cannot exclude the possibility that the SG formation *per se* might be altered in those cells due to the sequestration of essential SG-promoting factors in preformed dFMRP granules. The distribution of ΔKH, ΔRGG, and ΔpolyQ/N mutants in Schneider cells containing preformed dFMRP granules also did not undergo modification upon arsenite treatment, further suggesting that dFMRP cannot be transferred from dFMRP granules to SG (Fig. 5; panels 2–4. See also Videos S3, S4). However, and in contrast to GFP-dFMRP, we could not observe a clear change in the distribution of either mutant, e.g. in their recruitment to SG in cells lacking preformed dFMRP granules (Fig. 5. See also Videos S3, S4). Finally, we monitored motion of ΔPP mutant polypeptides which, as described above (Figs. 4E and S7), should accumulate in SG upon arsenite treatment. We found that arsenite induced ΔPP localization in SG as early as 15 min after treatment and peaking at 90 min (Fig. 5; panel 5 and Video S2). This formation of SG occurred in all analyzed ΔPP-expressing cells, thus validating the results obtained in fixed cells. These results suggest that the PP domain of dFMRP is dispensable for the recruitment of dFMRP in SG. Under these conditions, the KH domain, the RGG box as well as the C-terminal polyQ/N region of dFMRP seems to promote recruitment of the protein into SG in the present *Drosophila* cell model.

### FMRP Dynamics in SG

**Figure 6:**
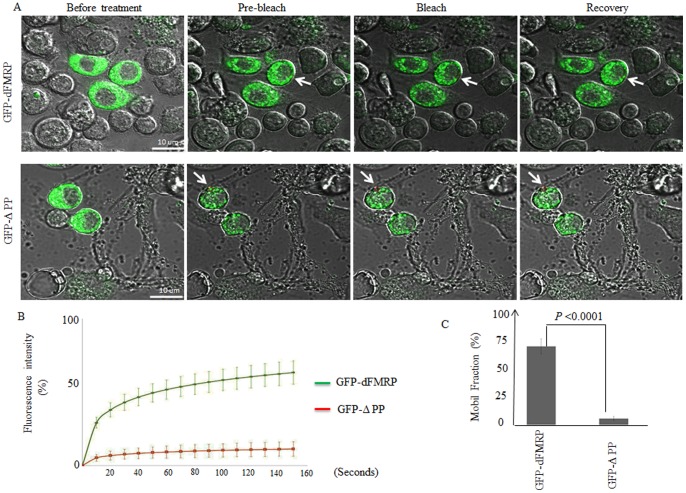
Analysis of GFP-dFMRP and GFP-ΔPP kinetics in SG by FRAP. (A–C) Schneider cells were transfected with either GFP-dFMRP or GFP-ΔPP and 48 h post transfections, SG were induced with arsenite (0.5 mM) for 1.5 h. A single SG (*red* circle; indicated by arrow) was photobleached and fluorescence recovery was recorded over 140 s using confocal microscopy. FRAP methodology is described in detail in “Materials and methods”. The indicated merged DIC and fluorescence images in (A) are selected for illustration. The recovery of dFMRP fluorescence in the photobleached area was quantified and plotted as a function of time, as indicated in (B). Curves are representative of 3 independent experiments with a total of 100 photobleached granules for each GFP fusion protein. (C) Bar graphs of MF for GFP-dFMRP and its ΔPP mutant are indicated with error bars corresponding to the SD of 3 independent experiments. The indicated *P-*values were calculated using an unpaired Student’s *t-*test (n = 3).

Previous studies have shown that RFP-dFMRP shuttles in and out neuronal granules [32]. Whether and how FMRP present in SG is in dynamic equilibrium with its free fraction is not known. To gain insight on the kinetics of FMRP trafficking between SG and the cytosol, and to determine the role of dFMRP functional domains in such trafficking, we relied upon fluorescence recovery after photobleaching (FRAP) experiments on GFP-dFMRP present in SG upon treatment of Schneider cells with arsenite. In this case, we chose cells lacking preformed dFMRP granules and selected granules that formed strictly only by arsenite treatment. Following the transfection of Schneider cells with various GFP-dFMRP constructs, individually formed SG upon arsenite treatment were bleached and allowed to recover over a period of 140 s. The intensity of recovering fluorescence was recorded every 5 s by confocal microscopy and plotted against time. The procedure was repeated twice to verify the reproducibility of recovery and the independence of percentage of recovery from the photobleached granules. With these experimental settings, GFP-dFMRP recovered up to 55±5% of unbleached intensity within 140 s (Fig. 6A–B). We obtained similar results after FRAP analysis of GFP-hFMRP present in SG of HeLa cells (Fig. S8). To validate these results we quantified the mobile fraction (MF), which provides a measure of the concentration of free molecules within the bleached area. The data show that the percentage of the MF for GFP-dFMRP correlates with the percentage of recovery recorded (Fig. 6C; *P*<0.04). Taken together, these results indicate that a significant fraction of total FMRP shuttles between SG and the cytosol. As described above (Figs. 4, 5 and S7), arsenite treatment induced an extensive localization of the ΔPP mutant within SG, indicating that dFMRP-protein interactions, which are mediated by the PP domain, might not contribute to promoting dFMRP recruitment in SG. Surprisingly, FRAP experiments showed that fluorescence of SG-associated ΔPP failed to recover after bleaching (Fig. 6A–B). This is consistent with the very low percentage (∼5%) of the MF recorded in SG found in that mutant (Fig. 6C), as compared to GFP-dFMRP (*P*<0.04). These results indicate that arsenite induced the recruitment of ΔPP in SG where it became stably sequestered. Thus, although dFMRP-protein interactions that are mediated by its PP domain seem to be dispensable for dFMRP recruitment in SG, these interactions are likely required for its shuttling between RNA granules and the cytosol.

## Discussion

Formation of RNA granules is critical for an adequate cellular response to external stimuli. While FMRP is known to be one of the major components of SG, the potential involvement of this protein in the regulation of SG formation across species is still unclear. Moreover, the determinants that are required for FMRP localization in SG as well as its dynamics in SG are still completely unknown. Using *Drosophila* cells as a model, we investigated the role of dFMRP in the formation of SG and defined its dynamics in SG. We found that either decreasing the amount of dFMRP in cell culture or deleting its function in ovaries does not prevent SG induction by stress. These results thus ruled out an essential function of this protein in SG formation in *Drosophila* cells tested, despite the fact that the protein is quantitatively recruited in SG. While both KH and RGG RNA-binding domains as well as the C-terminal polyQ/N region of dFMRP are required for its recruitment in SG, the PP domain seems to be dispensable for such activity. The latter domain is however required for dFMRP trafficking between SG and the cytosol. Our study suggests that FMRP-proteins interaction mediated by the PP domain is critical for dFMRP shuttling between RNA granules and the cytosolic dFMRP pool.

A previous study showed that FMRP deficiency reduces, but does not abolish the SG formation in *fmr1^−/−^* MEF upon arsenite treatment [22]. This decrease appears to affect the size rather than the number of SG, suggesting that FMRP does not play a key role during the initiation phase of SG formation. FMRP might instead induce the recruitment of additional factors into SG once these structures have started forming and/or promote SG stabilization. This idea is further supported by a genome-wide RNAi screen, which failed to identify FMRP among mammalian genes that are required for SG formation upon arsenite treatment [33]. Moreover, our results show that decreasing dFMRP level using siRNAs does not affect either the size or number of SG in Schneider cells upon stress (Fig. 2). To exclude the possibility that SG formation in dFMRP-depleted cells might have occurred due to residual dFMRP protein, we established a clonal system to analyze SG formation in ovaries harboring both dFMRP-knockout and dFMRP-expressing cells. Using this system, we show for the first time that SG formation occurs in either heat-shocked or arsenite-treated *Drosophil*a ovaries and that dFMRP deficiency does not prevent SG formation (Fig. 3). Future studies are needed however to determine if dFMRP could promote formation of SG in specific tissues. It is also possible that the role of FMRP in promoting SG might be crucial in higher organisms such as mammals but is dispensable in lower animals such as *Drosophila*. These results would also reflect biological differences in the mechanism of SG formation among organisms, as recently suggested between different yeast species [34] .

Although dFMRP has been shown to localize in SG years ago, the recruitment and dynamic aspects of this phenomenon had not previously been investigated. Live cells imaging showed that deletion of either the KH and the RGG domains of dFMRP prevented the recruitment of the protein in SG, suggesting that RNA-binding activity mediated by each RNA-binding domain of dFMRP is required for its localization in SG.

McKnight laboratory have recently developed a cell-free formation of RNA granules [35], [36]. Using this *in vitro* system, the authors found that trapping of RNA-binding proteins in granules occurs independently of RNA [35], [36]. The association of such RNA-binding proteins with *in vitro* assembled granules is mediated by low complexity sequences present within those proteins. The low complexity sequence of FMRP lies within the conserved RGG motif and promotes binding of FMRP to *in vitro* assembled granules [35], [36]. It is thus possible that the RGG domain of dFMRP induces trapping of the protein in SG through its low complexity sequence independently of RNA. The KH domain of FMRP lacks however such low complexity sequence, indicating that RNA binding activity mediated by the KH domain likely contributes together with the low complexity sequence present within RGG in inducing dFMRP trapping in granules *in vivo*. Recent studies described an aggregation prone role of Q/N-rich motifs in facilitating the localization of specific proteins in either PBs or SG [37], [38], [39]. In keeping with this, the C-terminal polyQ/N region of dFMRP seems also to be involved in dFMRP localization in SG, suggesting that the spliced variant of dFMRP lacking the C-terminal region might not be efficiently recruited to SG. The related C-terminal domain of human FMRP facilitates an interaction with kinesin motor protein and is needed for efficient FMRP-mediated dendritic RNA transport (Dictenberg et al., 2008), which could thus explain its role in promoting dFMRP transport into SG. This is consistent with a recent study describing the localization of mammalian kinesin proteins with SG [40]. However, this study suggested that dynein rather than kinesin motors are involved in SG formation [40]. The finding that kinesin is dispensable for formation of SG does not however exclude a possible role of kinesin proteins in promoting dFMRP localization in SG. Alternatively, it is possible that dynein rather than kinesin could facilitate association of dFMRP with SG. In any case, future experiments are needed to decipher the role of motor proteins in the association of dFMRP with SG. Our results also showed that the PP domain is dispensable for its recruitment in SG, suggesting that dFMRP-protein interactions that are mediated by the PP domain do not contribute to its localization in SG. Localization of this mutant to SG is likely to be mediated by the KH, RGG and the Q/N rich domain. Using FRAP, we demonstrate that dFMRP is in constant exchange between a “free” cytosolic pool and SG (Fig. 6). This property is not specific to *Drosophila* since we observed similar dynamic changes with mammalian FMRP (Fig. S8). Shuttling of dFMRP between SG and the cytosol was significantly reduced by deletion of PP domain, suggesting that protein interactions are required for dFMRP shuttling between the two compartments. At this stage, the identity of dFMRP partners that promote its trafficking between SG and cytosol are still unknown, and experiments are therefore underway to identify such proteins. Nevertheless, our results clearly suggest that the mechanisms controlling the recruitment of FMRP into SG and those that promote its shuttling between granules and the cytosol are uncoupled. To our knowledge, this is the first demonstration of the regulated shuttling activity of a SG component between RNA granules and the cytosol.

Estes and coll. had found that dFMRP rapidly shuttles between the naturally occurring neuronal RNA granules and the “free” cytosolic fraction [32]. The shuttling kinetics of mRFP-dFMRP in endogenous neuronal granules are similar to those described here for GFP-dFMRP in SG. It will be interesting to assess whether dFMRP shuttling to and from neuronal granules requires its PP domain. In their study, Estes et al. demonstrated that dFMRP shuttling promotes trafficking of its mRNA targets between RNA neuronal granules and the cytosolic fraction [32]. The mechanisms governing the promotion of mRNA trafficking by dFMRP are still unknown, as well as the relative efficiency of the latter process. Our investigations using SG predicts a working model in which FMRP binds to its mRNA targets and recruits them into RNA granules where they are incorporated in complexes whose dynamics are maintained by FMRP-protein interaction. Future experiments using SG should contribute to dissect the mechanism(s) by which FMRP-proteins interaction might control its shuttling activity; as well as the actual trafficking pathway of its associated mRNAs between RNA granules and the “free” cytosolic fraction. Posttranslational modifications of FMRP, such as phosphorylation and methylation, are known to regulate FMRP interactions with RNA, polysomes and proteins [14], [15], [41], [42], [43], [44], [45]. We hypothesize that such modifications are likely to play a key role in modulating FMRP and bound RNA shuttling between RNA granules and cytosol.

## Materials and Methods

### Cell Lines and Cultures

HeLa cervical cancer cells were obtained from the American Type Culture Collection (Manassas, VA; ATCC). Cells were cultured at 37°C in DMEM (Sigma-Aldrich, St. Louis, MO) supplemented with 10% fetal bovine serum (FBS), penicillin, and streptomycin (all supplements from Sigma-Aldrich). *Drosophila* Schneider cells were obtained from Dr. Robert Tanguay (Laval University) and were cultured at 25°C in Schneider medium (Sigma-Aldrich) supplemented with 10% FBS, penicillin and streptomycin.

### Antibodies

Phospho-specific anti-eIF2α was purchased from Cell Signaling Technology (Beverly, MA). Anti-deIF2α (EIF2S1) was obtained from Abcam (Cambridge, MA). Anti-dPABP [46] and anti-deIF4E [47] were kindly provided by Dr. Nahum Sonenberg (McGill University). Anti-dFMRP hybridoma (anti-dFMRP, 5B6-f) was obtained from Developmental Studies Hybridoma Bank (Iowa City, IA) and cultured as recommended by the manufacturer to produce anti-dFMRP antibodies.

### Small-interfering RNA (siRNA) Experiments

siRNA-dFMRP and non-targeting control siRNA were purchased from Dharmacon (Lafayette, CO). siRNA transfections were performed essentially as described [48], using HiPerFect reagent (Qiagen) following the manufacturer’s protocol. Twenty-four h before transfection, Schneider cells were plated on concanavalin A-treated coverslips 24 h before transfection at a density leading to 60–80% confluence at the moment of transfection. For a 6-well plate, annealed duplexes were used at a final concentration of 50 nM. Forty-eight h postransfection, cells were treated with siRNA (50 nM) for an additional 48 h. Cells were then either fixed and processed for immunofluorescence, or harvested for protein extraction. The sequences of the siRNAs used are:

siRNA-dFMRP-1∶5′-GGACAAGAGTGGCGTGTTT-3′

siRNA-dFMRP-2∶5′-GCAGAAGGCAGAAGAACAA-3′

### Immunofluorescence and RNA FISH

Following fixation and permeabilization (20 min in 3.7% paraformaldehyde at room temperature followed by a 15-min immersion in MeOH at −20°C), cells were incubated with primary antibodies diluted in 0.1% (v/v) Tween-20/PBS (PBST) for 2 h at room temperature. After rinsing with PBST, cells were incubated with goat anti-mouse/rabbit IgG (H+L) secondary antibodies conjugated with the Alexa Fluor dye of the appropriate maximum absorption wavelength (−405, −488 or −594) for 1 h, washed, and then mounted.

For FISH experiments, cells were first fixed in 3.7% paraformaldehyde for 20 min at room temperature, then permeabilized by a 15-min immersion in 0.1% Triton X-100/PBS. Poly(A)^+^ mRNAs were detected using a custom made 5′-tagged Alexa Fluor® 594-oligo [dT]_25_ (Invitrogen, Burlington, ON, Canada) diluted in PBS to a final concentration of 0.2 µM. Hybridization was performed by modifying the method presented in [49]. Briefly, cells were incubated with the oligo (dT)/PBS for 30 minutes at 42°C, then overnight at 37°C. Cells were then washed twice with 2X SSC (20 min at 37°C) followed by one wash with 0.5X SSC (20 min at 37°C), and finally with PBS.

After hybridization, cells were processed for immunofluorescence as described above. RNA and proteins were visualized using the LSM 700 confocal laser scanning microscope (Zeiss), equipped with a ZEN 2009 software for image acquisition and analysis. Images were acquired using the following settings: 63X oil objective (zoom 1.0), 0.06 µm for pixel size, and 1.00 airy units as pinhole.

### Induction of SG in *Drosophila* Ovaries

Ovaries were dissected in PBS and then transferred to Shields and Sang M3 insect medium supplemented with 2% fetal bovine serum and 2.5% fly extract. Stress granules were induced by incubating ovaries at 37°C for 3 h or addition of 0.5 mM arsenite for 1.5 h at 25°C. Where indicated, ovaries were pre-incubated for 0.5 h with 100 µg/ml cycloheximide or 200 µM puromycin, which were maintained throughout heat shock or arsenite treatment.

### Clonal Analysis

Flies were raised on standard food at 25°C. Homozygous mutant clones for *dfmr1* were produced in the follicular epithelium using the amorphic allele *dFMRP^Δ50M^* and the FLP/FRT system [50], [51]. Clonal analysis was performed in 3-d-old ♀ of the following genotype: w[*]; P{w[+mW.hs] = en2.4-GAL4}e22cP{w[+mC] = UAS-FLP1.D}JD1/+;P{ry[+t7.2] = neoFRT}82B ry [506]/P{ry[+t7.2] = neoFRT}82B, *dfmr1^Δ50M^*.

### Immunostaining of *Drosophila* Ovaries

Ovaries were heat fixed by placing them in E-wash (10 mM NaCl, 0.1% Triton-X-100) at 80°C, which was immediately cooled down by addition of ice-cold E-wash. Then, ovaries were incubated for 1 h in methanol at room temperature. Ovaries were saturated with 2% goat serum in 0.3% Triton-X-100/PBS (PBT) for 1 h, and incubated with primary antibodies at 4°C over-night. After washing with PBT, ovaries were incubated with secondary antibodies for 1 h at room temperature, washed again in PBT and finally mounted for confocal analysis.

### DNA Manipulation

To generate the vector pAc5.1/V5-HisA (Invitrogen) encoding GFP-dFMRP, total RNA was extracted from Schneider cells with the Omniscript Reverse Transcription kit (Qiagen) and used in a reverse transcription reaction to make dFMRP cDNA using oligo(dT). The reverse transcription product was then subjected to a PCR reaction using dFMRI-XhoI-F (5′-GGCCTCGAGCTATGGAAGATCTCCTCGTG-3′) and dFMR1-EcoRI-Rend (5′-GGCGAATTCTTAGGACGTGCCATTGAC-3′) in order to amplify the dFMRP cDNA. Amplified dFMRP cDNA was digested, purified and then incorporated into the digested (XhoI/EcoRI) pAc-GFP-C1 vector (Invitrogen) by ligation to generate GFP-dFMRP construct. GFP-dFMRP was then amplified by PCR using GFP-EcoRI-F oligo (5′-GGCGAATTCCGCCACCATGGTGAGCAA-3′) and dFMR1-EcoRI-Rend (5′-GGCGAATTCTTAGGACGTGCCATTGAC-3′). The PCR product GFP-dFMR1 was then digested at both ends with EcoRI and purified for insertion into the pAc5.1/V5-HisA *Drosophila* vector previously digested with EcoRI. pAc5.1/V5-HisA vectors encoding the GFP-dFMRP ΔKH, ΔRGG, and ΔPP variants were generated by ligation of PCR products amplified from pAc5.1/V5-HisA-GFP-dFMRP. The PCR products were first digested with the corresponding restriction enzymes whose sites are present in the primers used for PCR amplification before ligation. For the GFP-dFMRP-ΔPP mutant, GFP-EcoRI F and dFMRP-BamHI R342 oligos were used to amplify the first PCR fragment. Oligos used to amplify the second fragment were dFMRP-BamHI F664 and dFMRP-XbaI Rend. Both fragments were digested and joined to pAc5.1/V5-HisA previously digested with EcoRI and XbaI. For GFP-dFMRP-ΔKH mutant, the first PCR fragment was amplified with the GFP-EcoRI F and dFMRP-BamHI R672 oligos, and the second PCR fragment amplified with the dFMRP-BamHI F1012 and dFMRP-XbaI Rend. Amplified fragments were digested and ligated into pAc5.1/V5-HisA previously digested with EcoRI and XbaI. For the GFP-dFMRP-ΔRGG mutant, we used the GFP-EcoRI F with dFMRP-BamHI R1413 primers to amplify the first PCR fragment and the dFMRP-BamHI F1519 with dFMRP-XbaI Rend primers to amplify the second PCR fragment. The PCR fragments were digested and ligated into pAc5.1/V5-HisA that was digested with EcoRI and XbaI. The following primers were used: GFP-EcoRI-F, 5′-GGCGAATTCCGCCACCATGGTGAGCAA-3′; dFMRP-BamHI R342, 5′-GGCGGATCCCAGACGACCCAATTCACA-3′; dFMRP-BamHI F654, 5′-GGCGGATCCTACGTTGAGGAGTTCCGT-3′; dFMRPI-XbaI Rend, 5′-GGCTCTAGATTAGGACGTGCCATTGAC-3′, dFMRP-BamHI R672, 5′-GGCGGATCCCTCAACGTAGTTTCCACG-3′; dFMRP-BamHI F1012, 5′-GGCGGATCCCTGGCGCATGTACCCTTT-3′; dFMRP-BamHI R1413∶5′-GGCGGATCCGTTGTAGCCACGCTGCTG-3′; dFMRP-BamHI F1519∶5′-GGCGGATCCAACGATCAGCAGAATGGC-3′.

To generate the vector pAc5.1/V5-HisA encoding GFP-dFMRP-ΔpolyQ/N, total RNA was extracted from Schneider cells and used in a reverse transcription reaction to make cDNA using oligo(dT). The reverse transcription product was then subjected to a PCR reaction using dFMRI-XhoI-F (5′-GGCCTCGAGCTATGGAAGATCTCCTCGTG-3′) and dFMRP-R1520 (5′-GGCGAATTCTTAATCGTTGCGTGGCGG-3′). Amplified dFMRP cDNA was digested, purified and then incorporated into the digested (XhoI/EcoRI) pAc-GFP-C1 vector by ligation to generate GFP-dFMRP construct. GFP-dFMRP was then amplified by PCR using GFP-EcoRI-F oligo (5′- GGCGAATTCCGCCACCATGGTGAGCAA-3′) and dFMR1-EcoRI-R1520 (5′-GGCGAATTCTTAATCGTTGCGTGGCGG-3′). The PCR product GFP-dFMR1 was then digested at both ends with EcoRI and purified for insertion into the pAc5.1/V5-HisA *Drosophila* vector previously digested with EcoRI.

### DNA Transfection and Immunoprecipitation

For DNA transfection, Schneider cells were transfected with 0.5 µg of DNA in a 6-well plate using the Effectene transfection reagent kit (Qiagen). For immunoprecipitation, cells were collected and lysed at 4°C with lysis buffer (50 mM Tris-HCl, pH 7.4; 0.5% NP-40; 150 mM NaCl; 1 mM MgCl_2_; 0.25 mM phenylmethanesulfonylfluoride; 0.5 mM DTT) containing a cocktail of protease inhibitors (Roche, Laval, QC, Canada) and 40 U/µl RNase Inhibitor (Invitrogen). The extract was then incubated with protein A Sepharose CL-4B beads (GE Healthcare Life Sciences, QC, Canada) conjugated with the appropriate antibody. Following three washes with lysis buffer, proteins were eluted by resuspending the beads with an equal volume of loading dye buffer. Five percent of the suspension was used for immunoblot analysis of the immunoprecipitated proteins.

### Polysome Preparation

Polysomes were prepared as follows. Schneider cells were collected in lysis buffer (20 mM Tris-HCl pH 7.4, 1.25 mM MgCl_2_, 150 mM NaCl, 1 mM DTT, 1% NP-40, and 5 U/ml of RNase inhibitor (Invitrogen) supplemented with complete Mini EDTA-free Protease Inhibitor Cocktail tablets (Roche). The cell homogenate was then clarified by centrifugation at 12,000 rpm for 10 min at 4°. The cytoplasmic extract was then loaded onto a 15% to 55% linear sucrose gradient (wt/vol) previously generated with an Isco Model 160 gradient former (Teledyne Isco, Lincoln, NE) and then separated by sedimentation velocity through centrifugation for 2.5 h at 37,000 rpm using a Sorvall ultracentrifuge rotor TH-641 (Du Pont, DE, USA) at 4°C. The sucrose gradient was processed for fractionation using an Isco type 11 Optical Unit with 254 nm and 280 nm filter set (Teledyne Isco). Equal fractions were collected with continuous monitoring of absorbance at 254 nm using an Isco UA-6 UV-vis detector (Teledyne Isco). Fractions were precipitated, resuspended in an equal volume of SDS-PAGE sample buffer and analysed by western blot.

### Imaging and FRAP

Images were acquired on a Zeiss LSM 700 confocal system (Zeiss). For live cell imaging and SG formation monitoring, acquisitions using the 488-nm line at 2% and differential interference contrast (DIC) mode were taken before and after arsenite treatment. Using the same parameters, videos were acquired by taking images every 3 min for 90 min and merging them side to side. For FRAP, a single GFP-labeled granule per cell was photobleached using the Photo Bleach function of the Zeiss LSM 700 imaging system with the diode laser 488-line set at 100%. The acquisition of recovery time points was done using the laser 488-line set at 2%. A first picture was taken before FRAP and then, pictures were continuously taken during 30 cycles. Each picture shot required an average of 5 s, depending of the size of the photobleached region, for a total time of approximately 140 s. The FRAP analysis included the determination of the average fluorescence intensity of a region of interest containing an unbleached granule as well as an area of background fluorescence. To ensure that the bleaching laser did not damage the cell, the same granules were photobleached, and fluorescence recovery was recorded again. Measurements of fluorescence were done using imaging ZEN software (Zeiss). Briefly, background fluorescence was subtracted from the bleached and unbleached granules and recovery fluorescence values were normalized to a percentage of original fluorescence. The bleached granule was then corrected to the fluorescence of the unbleached granule to adjust for slight changes in focus or slight time-dependent bleaching. Recovery could then be compared in multiple granules of different sizes and from different cells across multiple experimental sessions. Mobile fraction (MF) measurements (i.e. the percentage of fluorescence proteins capable of diffusing into a bleached region of interest during the time course of the experiment) were determined using ZEN software (Zeiss).

## Supporting Information

Figure S1 **Colocalization of dFMRP with poly(A)^+^ mRNA.** Schneider cells were treated with arsenite (0.5 mM; 1.5 h), fixed, permeabilized, and then incubated with 0.2 µM of an Alexa Fluor 594-labeled oligo(dT) probe to detect poly(A)^+^ mRNA (*red* signal in merged pictures). SG were detected using anti-dFMRP antibodies (*green* signal in merged pictures). The percentage of cells harboring SG (>3 granules/cell) is indicated in the merged pictures. Representative results from 5 different fields and 3 different experiments containing a total of 1,000 cells are shown. Scale bars are shown.
(TIF)

Figure S2 **SG depolymerize during recovery from stress.** Schneider cells were treated with arsenite (0.5 mM) or incubated under heat shock conditions (37°C) for 1.5 h. Cells were then washed with PBS and allowed to recover from arsenite treatment for 2 h in arsenite-free medium. Heat-shocked cells were let to recover at 25°C for 2 h. Cells were then fixed and processed for immunofluorescence as described above. dFMRP is detected as green signal and blue staining is for DAPI. The indicated percentage of cells harboring SG was calculated as in Fig. 1.
(TIF)

Figure S3 **Reducing dFMRP levels does not affect localization of the SG marker GFP-deIF4A in SG.** Schneider cells were first treated with non-specific or dFMRP-directed siRNA-1 for 48 h then were transfected with GFP-deIF4A for an additional 48 h. Following transfection, cells were treated with arsenite (0.5 mM) for 1.5 h. Cells were then processed for confocal microscopy to detect GFP-deIF4A in SG (*green*). Depletion of dFMRP was assessed using specific antibodies as described in Fig. 1. The indicated percentage of cells harboring SG was calculated as in Fig. 1.
(TIF)

Figure S4 **Treatment of Schneider cells with cycloheximide but not with puromycin prevents SG formation.** (A–B) Cells were treated with either cycloheximide (100 µg/ml) or puromycin (200 µg/ml) for 0.5 h then were incubated under heat shock conditions for an additional for 1.5 h, in presence of cycloheximide and puromycin, respectively. Cells were then fixed and processed for immunofluorescence to detect the SG marker dFMRP (green signal). The indicated percentage of cells harboring SG was calculated as described above. Scale bars are shown.
(TIF)

Figure S5 (A) Treatment with puromycin does not inhibit formation of SG in either heat-shocked or arsenite-treated ovaries. Ovaries isolated from WT flies were treated with puromycin (200 µg/ml) for 0.5 h then were either heat-shocked at 37°C for 3 h or incubated with 0. 5 mM arsenite for 1.5 h, in presence of puromycin. Ovaries were then fixed, permeabilized and processed for immunofluorescence as described in “Materials and methods”. SG were visualized using bot anti-dFMRP and anti-dPABP antibodies. Scale bars are shown. (B) Surface view of the epithelium of a wild type ovariole (panels 1–2) or an ovariole in which dFMRP mutant clone was induced (panels 3–4) and stained for dFMRP and DAPI. Arrow points to a dFMRP mutant clone in a stage 8 follicle. In panels 3 and 4, nucleus of nurse cells, located underneath the follicular epithelium, are visible. Scale bars are shown.
(TIF)

Figure S6 **Colocalization of deIF4E with dFMRP granules.** Schneider cells were transfected with either GFP or GFP-dFMRP constructs for 48 h. Cells were then fixed and then processed for immunofluorescence to detect GFP or GFP-dFMRP (*green*). The intracellular localization of endogenous deIF4E (*red*) is revealed using antibodies specific to deIF4E. Scale bars are indicated.
(TIF)

Figure S7 **Colocalization of deIF4E with dFMRP granules under stress conditions.** Schneider cells were transfected with either GFP or GFP-dFMRP constructs for 48 h. Cells were then treated with arsenite (0.5 mM; 1.5 h), fixed and processed for immunofluorescence to detect deIF4E using specific antibodies (red signal). GFP and GFP-dFMRP are detected as green fluorescence. Scale bars are indicated.
(TIF)

Figure S8 **Dynamics of GFP-hFMRP in SG by FRAP.** (A–B) HeLa cells were transfected with GFP-hFMRP. Forty-eight h posttransfection, cells were treated with arsenite for 0.5 h. A single SG (*red* circle; indicated by arrow) was photobleached (A) and fluorescence recovery was recorded over 140 s (B) using confocal microscopy as described in Fig. 6. Scale bars are indicated.
(TIF)

Video S1 **Motion of GFP-dFMRP in SG as visualized in live cells.** Schneider cells were transfected with GFP-dFMRP fusion protein expression vector. After 48 h, arsenite (0.5 mM) was added and images were immediately recorded at 3-min intervals during 1.5 h. Time stamps are indicated on each video.
(AVI)

Video S2 **Motion of GFP-ΔPP in SG as visualized in live cells.** Schneider cells were transfected with GFP-ΔPP fusion protein expression vector. After 48 h, arsenite (0.5 mM) was added and images were immediately recorded at 3-min intervals during 1.5 h. Time stamps are indicated on each video.
(AVI)

Video S3 **Motion of GFP-ΔKH in SG as visualized in live cells.** Schneider cells were transfected with GFP-ΔKH fusion protein expression vector. After 48 h, arsenite (0.5 mM) was added and images were immediately recorded at 3-min intervals during 1.5 h. Time stamps are indicated on each video.
(AVI)

Video S4 **Motion of GFP-ΔRGG in SG as visualized in live cells.** Schneider cells were transfected with GFP-ΔRGG fusion protein expression vector. After 48 h, arsenite (0.5 mM) was added and images were immediately recorded at 3-min intervals during 1.5 h. Time stamps are indicated on each video.
(AVI)
