# Supplementary figures and images for: Characterization of Fragile X Mental Retardation Protein Recruitment and Dynamics in Drosophila Stress Granules
Source: PLoS One. 2013 Feb 7;8(2):e55342. doi: 10.1371/journal.pone.0055342 (PMC3567066; doi:10.1371/journal.pone.0055342)

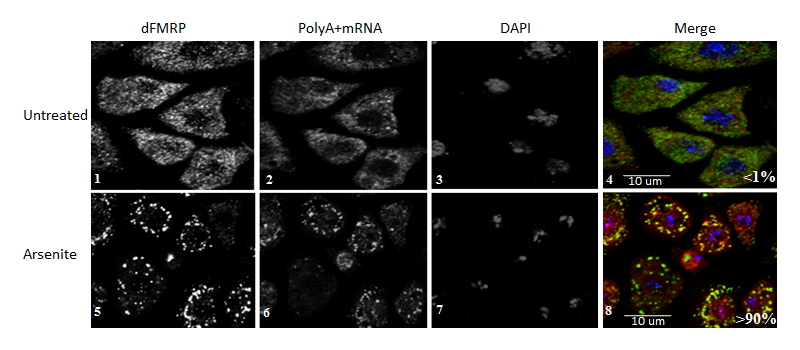

Supplement: Figure S1 — Colocalization of dFMRP with poly(A)+ mRNA. Schneider cells were treated with arsenite (0.5 mM; 1.5 h), fixed, permeabilized, and then incubated with 0.2 µM of an Alexa Fluor 594-labeled oligo(dT) probe to detect poly(A)+ mRNA (red signal in merged pictures). SG were detected using anti-dFMRP antibodies (green signal in merged pictures). The percentage of cells harboring SG (>3 granules/cell) is indicated in the merged pictures. Representative results from 5 different fields and 3 different experiments containing a total of 1,000 cells are shown. Scale bars are shown. (TIF) [file pone.0055342.s001.tif]

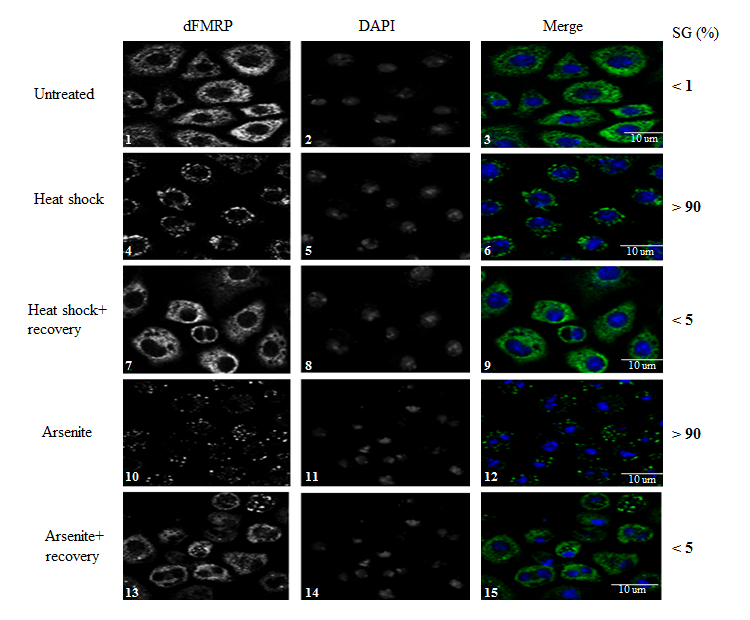

Supplement: Figure S2 — SG depolymerize during recovery from stress. Schneider cells were treated with arsenite (0.5 mM) or incubated under heat shock conditions (37°C) for 1.5 h. Cells were then washed with PBS and allowed to recover from arsenite treatment for 2 h in arsenite-free medium. Heat-shocked cells were let to recover at 25°C for 2 h. Cells were then fixed and processed for immunofluorescence as described above. dFMRP is detected as green signal and blue staining is for DAPI. The indicated percentage of cells harboring SG was calculated as in Fig. 1. (TIF) [file pone.0055342.s002.tif]

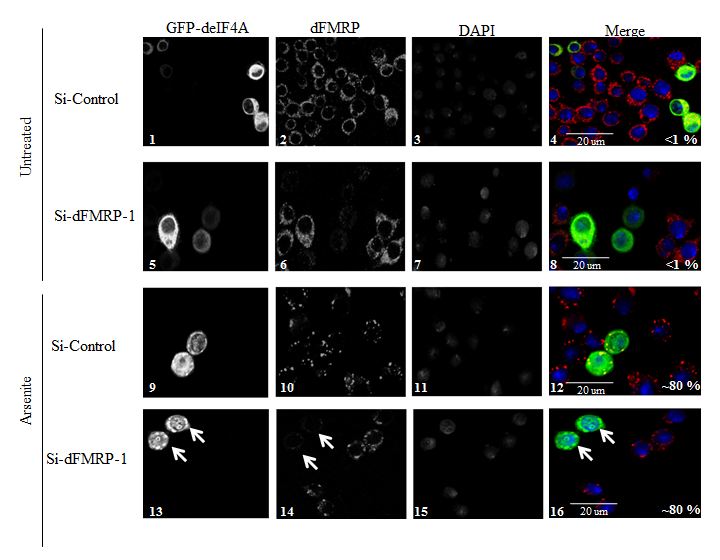

Supplement: Figure S3 — Reducing dFMRP levels does not affect localization of the SG marker GFP-deIF4A in SG. Schneider cells were first treated with non-specific or dFMRP-directed siRNA-1 for 48 h then were transfected with GFP-deIF4A for an additional 48 h. Following transfection, cells were treated with arsenite (0.5 mM) for 1.5 h. Cells were then processed for confocal microscopy to detect GFP-deIF4A in SG (green). Depletion of dFMRP was assessed using specific antibodies as described in Fig. 1. The indicated percentage of cells harboring SG was calculated as in Fig. 1. (TIF) [file pone.0055342.s003.tif]

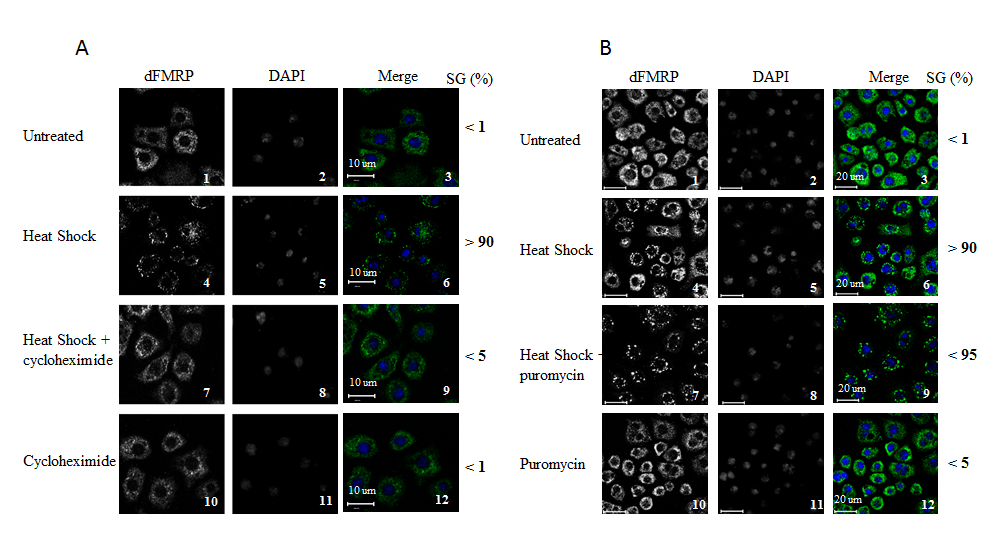

Supplement: Figure S4 — Treatment of Schneider cells with cycloheximide but not with puromycin prevents SG formation. (A–B) Cells were treated with either cycloheximide (100 µg/ml) or puromycin (200 µg/ml) for 0.5 h then were incubated under heat shock conditions for an additional for 1.5 h, in presence of cycloheximide and puromycin, respectively. Cells were then fixed and processed for immunofluorescence to detect the SG marker dFMRP (green signal). The indicated percentage of cells harboring SG was calculated as described above. Scale bars are shown. (TIF) [file pone.0055342.s004.tif]

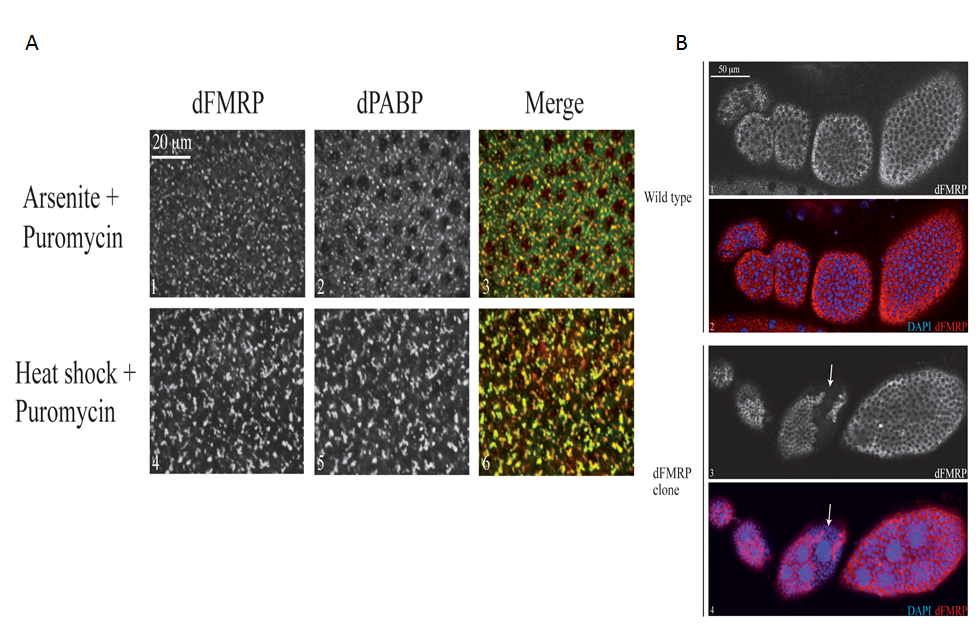

Supplement: Figure S5 — (A) Treatment with puromycin does not inhibit formation of SG in either heat-shocked or arsenite-treated ovaries. Ovaries isolated from WT flies were treated with puromycin (200 µg/ml) for 0.5 h then were either heat-shocked at 37°C for 3 h or incubated with 0. 5 mM arsenite for 1.5 h, in presence of puromycin. Ovaries were then fixed, permeabilized and processed for immunofluorescence as described in “Materials and methods”. SG were visualized using bot anti-dFMRP and anti-dPABP antibodies. Scale bars are shown. (B) Surface view of the epithelium of a wild type ovariole (panels 1–2) or an ovariole in which dFMRP mutant clone was induced (panels 3–4) and stained for dFMRP and DAPI. Arrow points to a dFMRP mutant clone in a stage 8 follicle. In panels 3 and 4, nucleus of nurse cells, located underneath the follicular epithelium, are visible. Scale bars are shown. (TIF) [file pone.0055342.s005.tif]

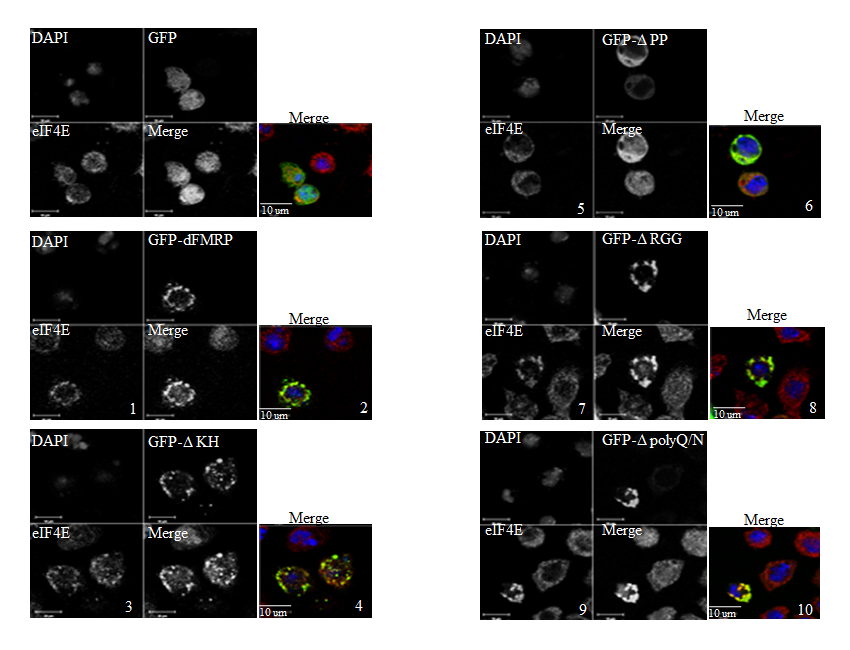

Supplement: Figure S6 — Colocalization of deIF4E with dFMRP granules. Schneider cells were transfected with either GFP or GFP-dFMRP constructs for 48 h. Cells were then fixed and then processed for immunofluorescence to detect GFP or GFP-dFMRP (green). The intracellular localization of endogenous deIF4E (red) is revealed using antibodies specific to deIF4E. Scale bars are indicated. (TIF) [file pone.0055342.s006.tif]

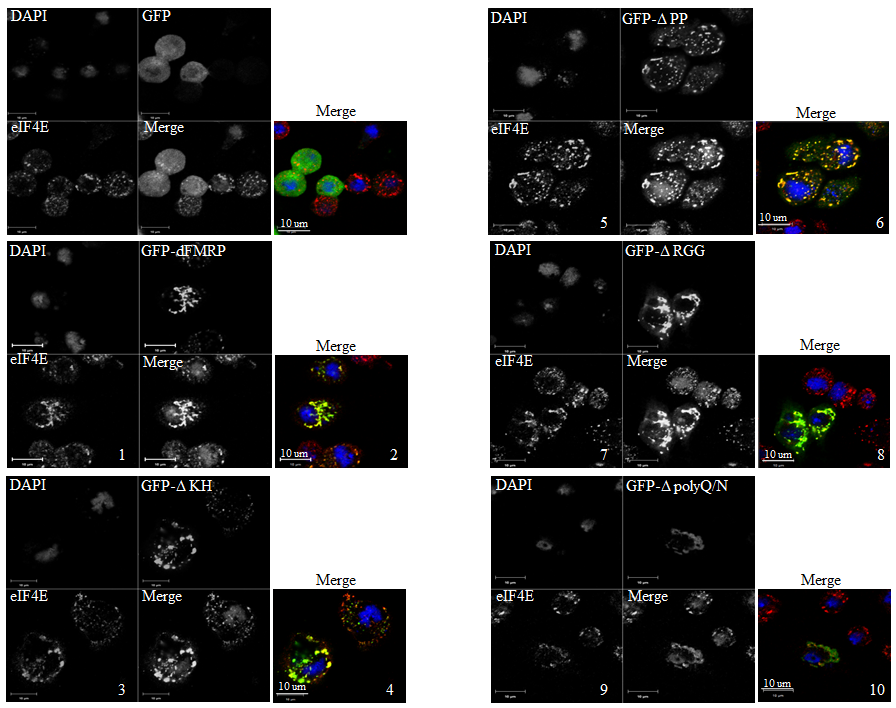

Supplement: Figure S7 — Colocalization of deIF4E with dFMRP granules under stress conditions. Schneider cells were transfected with either GFP or GFP-dFMRP constructs for 48 h. Cells were then treated with arsenite (0.5 mM; 1.5 h), fixed and processed for immunofluorescence to detect deIF4E using specific antibodies (red signal). GFP and GFP-dFMRP are detected as green fluorescence. Scale bars are indicated. (TIF) [file pone.0055342.s007.tif]

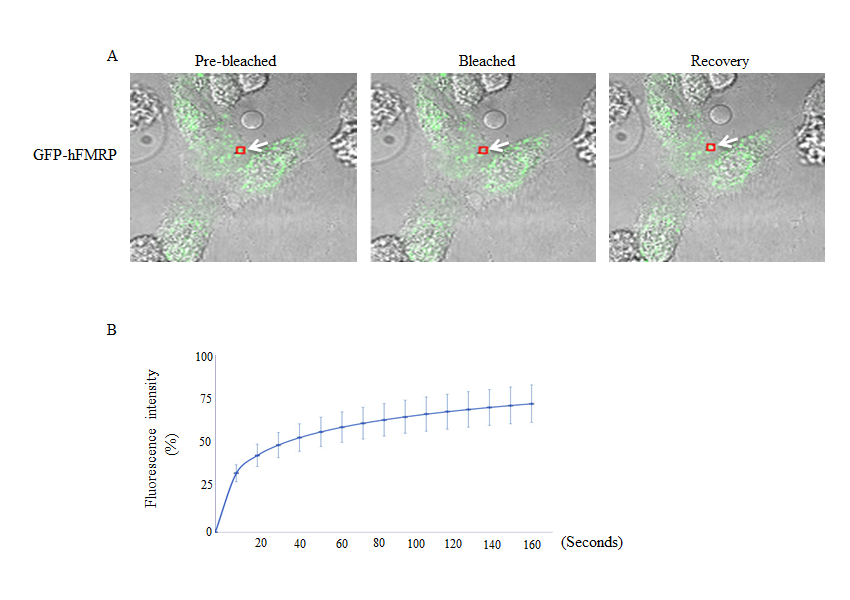

Supplement: Figure S8 — Dynamics of GFP-hFMRP in SG by FRAP. (A–B) HeLa cells were transfected with GFP-hFMRP. Forty-eight h posttransfection, cells were treated with arsenite for 0.5 h. A single SG (red circle; indicated by arrow) was photobleached (A) and fluorescence recovery was recorded over 140 s (B) using confocal microscopy as described in Fig. 6. Scale bars are indicated. (TIF) [file pone.0055342.s008.tif]
